# The Pseudoscience of Phrenology: A Historical Examination

**DOI:** 10.7759/cureus.113746

**Published:** 2026-07-31

**Authors:** Luke Bauerle

**Affiliations:** 1 College of Medicine, Medical University of South Carolina, Charleston, USA

**Keywords:** cerebral localization, cortical localization, gall, gender, neuroscience, phrenology, race, spurzheim

## Abstract

Among the numerous theories and concepts advanced throughout the history of medicine, phrenology remains one of the most recognizable and controversial. Promoted as a scientific discipline that linked human behavioral traits to scalp morphology, phrenology, from its inception by Franz Joseph Gall (1758-1828) and Johann Gaspar Spurzheim (1776-1832), faced significant criticism for its lack of reproducible evidence and its historical use in supporting racial and misogynistic ideologies. Despite its reputation for reinforcing social and racial stereotypes, some minority groups and social movements in the 19th century regarded phrenology as a systematic approach to self-reflection and empowerment within paternalistic and racially divided societies. Phrenological examination was viewed as a means of identifying personal flaws and pursuing self-improvement to contribute to one's community or enhance social status. Notably, the central theory of cerebral localization in phrenology stimulated subsequent research into the functions and structures of specific brain regions, influencing the development of neuroscience and medicine. As a result, later research acknowledged the validity of certain core concepts from phrenology, illustrating the evolving nature of scientific understanding.

This review article explores the history and societal utilization of phrenology in 19th-century America and Europe based on a review of literature from Google Scholar and PubMed databases. While acknowledging the contributions of the field's founders to our modern understanding of brain function, this article will also critically examine the flaws in the field's underlying theories as well as both the vindictive and beneficial applications of the field in numerous social issues of the early 19th century, thus illustrating the complex journey of once-disregarded scientific theories and proposed concepts.

## Introduction and background

Phrenology, as a scientific field, sought to link various human behavioral traits to scalp morphology, with its followers believing that certain traits corresponded to specific brain areas and that their activity exerted a mechanical force on the skull [1]. Though the field is now considered the epitome of fraudulent pseudoscience, its failures prompted detailed scientific investigations in brain science that would lead to the groundbreaking neuroscientific discoveries of the 19th century, making its founders influential figures in the history of neuroscience. Though now largely debunked, some of the concepts that defined the field continue to be debated today, particularly the notion of cerebral localization and the possible compartmentalization of the brain’s innumerable functions rather than their widespread cortical participation. Thus, it could be argued that phrenology did contribute to aiding humanity’s quest to understand the intricacies of the human brain and behavior.

## Review

Gall and the theory of cerebral localization

Though the notion of cortical localization, or the idea that different parts of the brain could be responsible for different functions and behaviors, was first proposed in Antiquity, it was Franz Joseph Gall (1758-1828, Figure 1) who would go on to propose his own theory of cortical localization in the late 1700s, a theory he likely carried with him from a young age. Gall recounted that some of his classmates during his youth, with superior memory for verbal material, often had bulging eyes, leading him to deduce that a specific area in the frontal lobes of the human brain devoted to verbal memory must exist in these students based solely on this physical feature [2]. By expanding this line of thinking into other aspects of human thought and cognition, Gall believed that it was not unreasonable to suspect that other regions, or "organs," of the brain could be specific to individual mental functions. Though Gall was not the first to propose this idea of specific tasks assigned to specific regions of the brain, it was Gall who argued that these organs could exert a force on an individual's skull, producing bumps and depressions [3]. More importantly, it was Gall who proposed that the results of this force could indicate an increase or decrease in a particular human quality, such as vanity, submissiveness, or destructiveness, which could be assessed by cranial palpation and detailed measurements.

**Figure 1:**
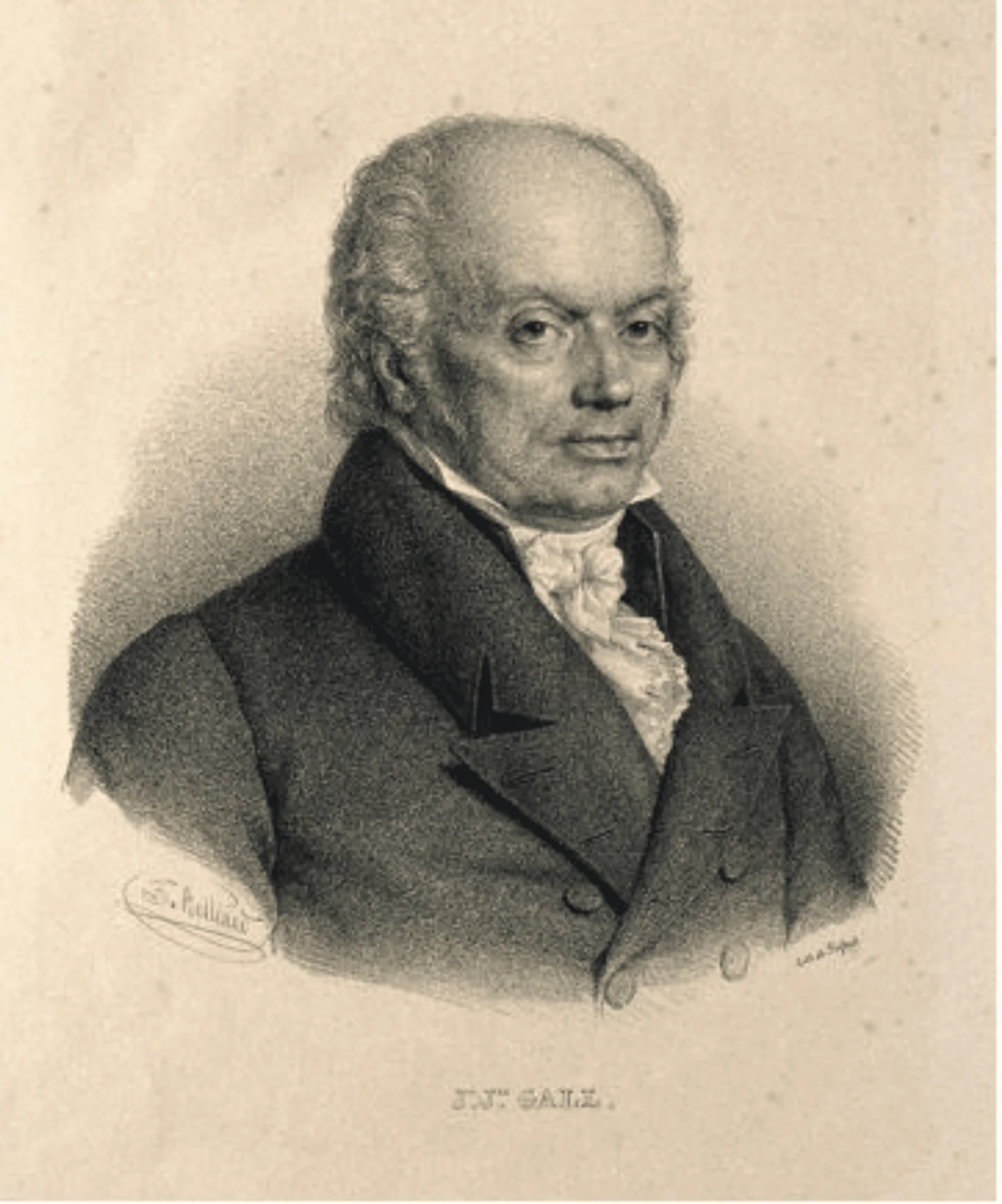
Franz Joseph Gall (lithograph by Z. Belliard, 1834) Image Credit: Wellcome Collection. Public Domain Mark. Licensed under Creative Commons Attribution 4.0 International (CC BY 4.0): https://wellcomecollection.org/search/images?query=q6dzq64a

For years in the late 1790s and early 1800s, Gall would travel throughout Europe, giving lectures to the public and academia on his theory, which he termed "organology," and examining the crania of individuals from both ends of the societal spectrum, from writers and scientists to criminals and the physically handicapped [3]. During these examinations, he would often note the location of a particular prominence or depression that likely corresponded with a person's most noteworthy talent or deficiency, from which he would make a cast of a person's skull with their determined cranial features and traits for future reference [3]. Following his work during this time, he theorized that the human psyche was composed of twenty-seven total aspects of personality, or "faculties," each with its own corresponding cortical and cranial location [2]. Though nineteen of these traits, such as affection, destructiveness, and desire of possession, were thought to be present in animals as well as humans, eight of the higher faculties, such as wisdom, satire, and poetic talent, were postulated to be uniquely human features that were all localized to the frontal lobes [4].

Regarding supposed evidence for this theory, Gall would often simply link a physical feature of a subject's skull with a particular action or lifestyle choice from that person's history and compare it to other patients with similar physical features and histories. For example, the organ for "carnivorous instinct" or "tendency to murder" was localized above the ears because Gall noticed a particular prominence in roughly the same location when he analyzed the skulls of carnivorous animals compared to herbivores, a student who was fond of torturing animals, a public executioner, and a butcher [3]. Another example was Gall's localization of the organ of sexuality in the cerebellum, with one of his sources of supposed evidence being the examination of a nymphomaniac widow [5]. Despite his faith in his doctrine of the mind, Gall never truly argued that he knew the anatomical boundaries of each of his proposed organs, thereby likely weakening the case for the accuracy of his conclusions and hindering the replicability of his theory [3].

Despite these issues, Gall published his work, Anatomy and Physiology of the Nervous System in General and the Brain in Particular, from 1810 to 1819 in five volumes, meticulously describing the results of his neuroanatomical studies and the theory behind his identified faculties [6]. Despite the public’s apparent popularity in the field, the scientific establishment never truly accepted the man or his theory as credible. By the time of Gall's death in 1828, the academic world had nearly disavowed the field, as it does today. However, though the field's original discipline had disappeared, another had arisen to become the new voice of the field, subsequently molding its concepts and practices into his own version, which would come to be embraced by many laypeople in 19th-century Europe and America.

Spurzheim and the popularization of phrenology

**Figure 2:**
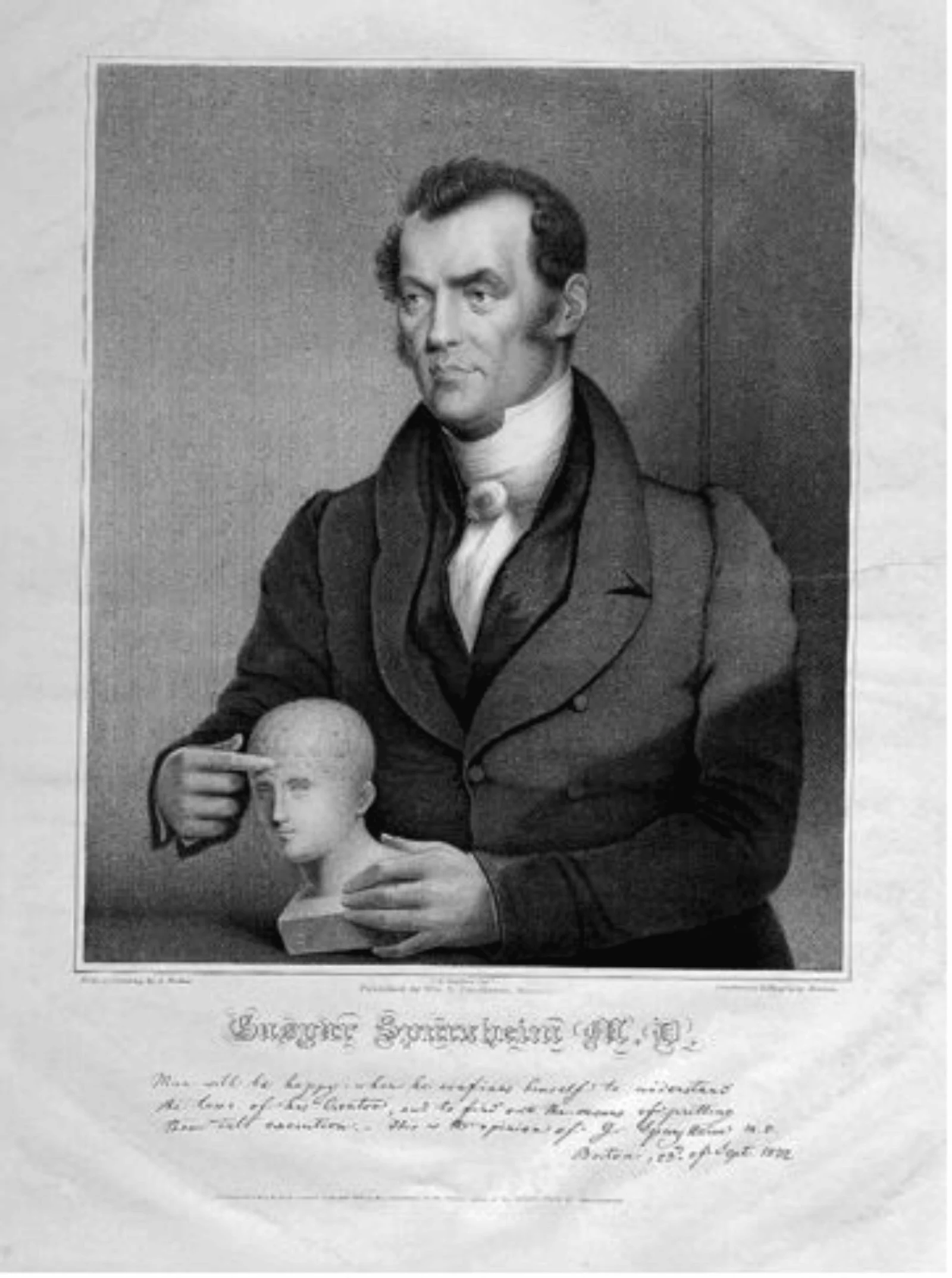
Johann Gaspar Spurzheim (lithograph by J.H. Bufford after A. Fisher, 1832) Image Credit: Wellcome Collection. Public Domain Mark. Licensed under Creative Commons Attribution 4.0 International (CC BY 4.0): https://wellcomecollection.org/search/images?query=y3z6296d

**Figure 3:**
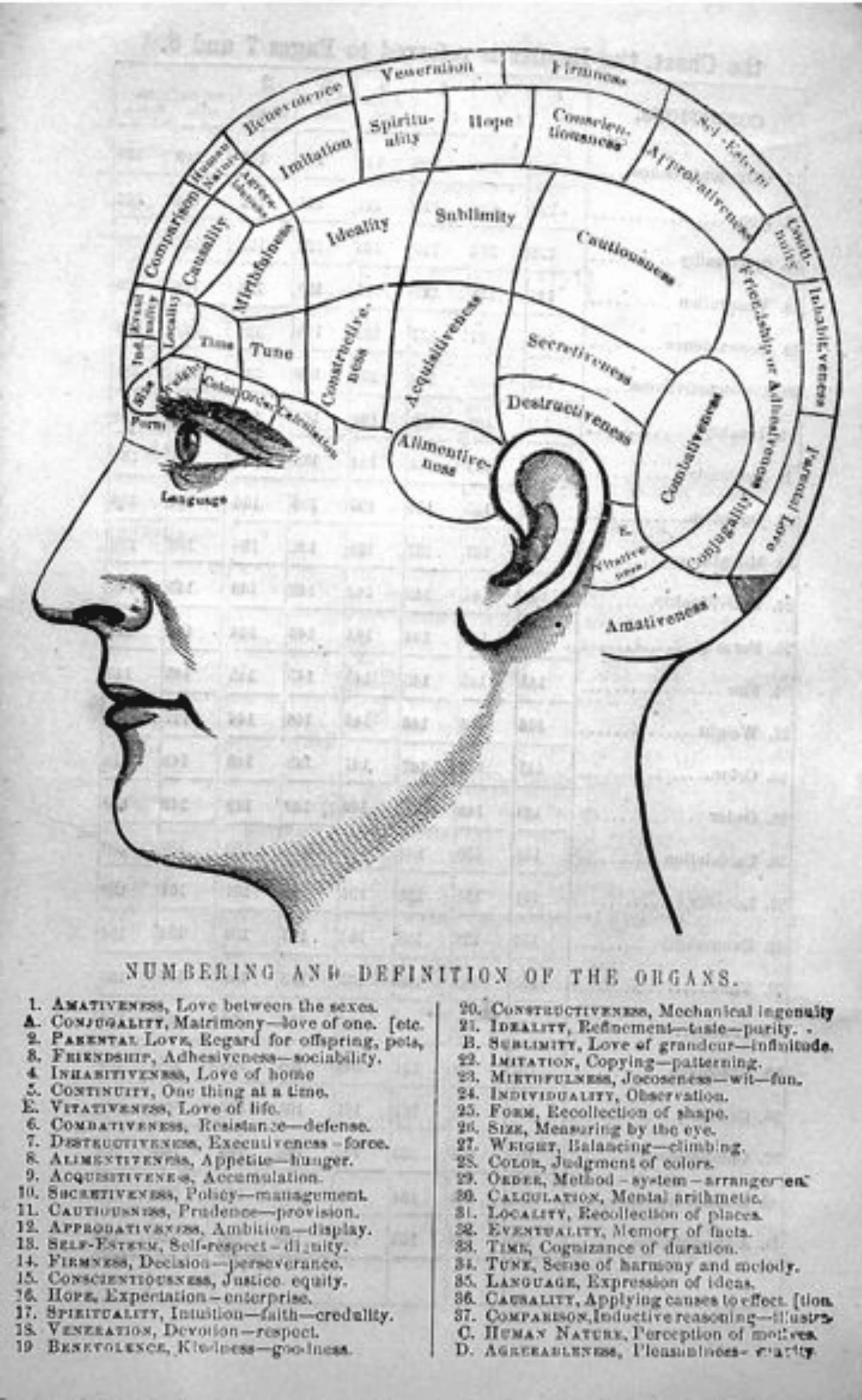
Phrenology head chart with numbering and definition of organs (illustration by O. S. and L. N. Fowler, 1859) Image Credit: Wellcome Collection. Public Domain Mark. Licensed under Creative Commons Attribution 4.0 International (CC BY 4.0): https://wellcomecollection.org/search/images?query=b5q4kemp

Though it was Gall who established the field, it was Johann Gaspar Spurzheim (1776-1832; Figure 2) who gave the field its now well-known name of "phrenology," or "study of the mind" [7,8]. Fueled by his interest in medicine, Spurzheim, much like Gall, was intrigued by a possible link between cranial features and mental attributes from an early age [9]. While studying medicine in Vienna, Spurzheim met his future mentor during a lecture by Gall himself [10]. After an exchange of ideas, Spurzheim eventually became Gall's pupil, aiding the pair's comparative anatomical studies and brain dissections conducted in European lecture halls before eager young medical students [9]. Despite this apparent fluid partnership, differences in ideology quickly emerged, particularly over the number and nature of phrenology's organs. Instead of Gall's 27 functional organs, Spurzheim declared that there were actually thirty-five total organs (Table 1, Figure 3) [9]. To Spurzheim, if a man became a murderer, it was because he chose to display the mental capabilities of a murderer instead of a group of organs inherited at birth deciding for him, as Gall would think. From these decisions, these organs would exert pressure onto the cranium to produce the prominences of the skull, which the phrenologist could then palpate and analyze. Eventually, this disagreement would lead Spurzheim to remove the supposed unpleasant faculties emphasized by Gall in his later works, prompting Gall himself even to criticize the work of his former pupil [11].

**Table 1:** Comparison of Gall and Spurzheim's proposed "organs" or "faculties" [1,12-14]

Number	Franz Joseph Gall (names/descriptions)	Johann Gaspar Spurzheim (names/descriptions)
1	Impulse to propagation/instinct of reproduction	Destructiveness/tendency to destroy
2	Tenderness for the offspring/parental love	Amativeness/physical love
3	Friendly attachment/affection	Philoprogenitiveness/parental love
4	Valor/self-defense/courage	Adhesiveness/friendly attachment
5	Carnivorousness/tendency to murder	Inhabitiveness/desire of permanence
6	Guile/acuteness/cleverness	Combativeness/tendency to fight
7	Larceny/sense of property	Secretiveness/tendency to restrain emotions and ideas
8	Pride/arrogance	Acquisitiveness/desire to possess and accumulate
9	Ambition/vanity/love of glory	Constructiveness/tendency to build
10	Circumspection/forethought	Cautiousness/desire to shun danger
11	Aptness to receive an education/memory of facts	Approbativeness/desire to praise, fame, or glory
12	Locality/sense of places	Self-esteem/self-respect, personal dignity
13	Recollection of persons/memory of people	Benevolence/desire of compassion, universal charity
14	Faculty for words/verbal memory	Veneration/religious emotion
15	Speech/sense of language	Firmness/determination, perseverance
16	Disposition for coloring	Conscientiousness/desire for justice, respect for rights
17	Sense for sounds/musical talent	Hope/tendency to expect good fortunes
18	Arithmetic, counting, time/sense of connectness between numbers	Marvellousness/desire for novelty
19	Mechanical skill/talent for architecture	Ideality/poetic talent
20	Comparative perspicuity/sagacity	Mirthfulness/wit
21	Metaphysical perspicuity/sense of metaphysics	Imitation/tendency to mimic
22	Wit, causality/sense of satire	Individuality/awareness of occurrences and events
23	Poetic talent	Configuration/recognition of external features of objects
24	Moral sense/good-nature/compassion	Size/perception of space, dimensions, and distance
25	Mimic/faculty to imitate	Weight and resistance/perception of momentum, weight, and resistance
26	Theosophy/sense of God and religion	Coloring/perception of colors and their harmonies
27	Perseverance/firmness/constancy	Locality/sense of relative position
28	-	Order/desire for physical arrangement
29	-	Calculation/talent for arithmetic
30	-	Eventuality/awareness of occurrences and events
31	-	Time/perception of time duration
32	-	Tune/musical talent
33	-	Artificial language/speech
34	-	Comparison/tendency to discover analogies, resemblances, and differences
35	-	Causality/relation of cause and effect

Despite their differences in ideology, the two men demonstrated their research and anatomical dissections in lecture halls in Austria, Germany, and France to great success, with one anatomist in particular noting that Gall's dissections were "more than I thought a man could discover in his whole life" [9]. Despite innovations in their dissection method and the general well-reception of their ideas by scientists and laypeople alike, Gall and Spurzheim's disagreements over the central principles of phrenology eventually caught up with them, leading to the pair's falling out and the eventual cessation of their collaboration in 1813 [15]. Following this split, Spurzheim set out to make a name for himself in the ever-growing phrenology movement by first traveling to Britain to deliver a series of lectures on the field. Overall, Spurzheim's talks were well received by both the public and the medical community, with one letter published in The British Journal denouncing those who ridiculed phrenology by simply stating that "…in a word, phrenology is a great fact…" [16]. While in Britain, he appeared to legitimize the field of phrenology to a certain extent within the scientific community, so much so that he was actually given a licentiate’s diploma by the Royal College of Physicians of London [2]. Following years of lectures and demonstrations, Spurzheim eventually traveled to the United States in 1832, where he gave talks in the lecture halls of the country’s most prestigious institutions, such as Yale and Harvard [2]. Despite his unexpected death in Boston that same year from typhoid fever, his version of phrenology would live on for nearly a century and would become popular throughout the world in many different realms of thought.

Social applications of phrenology

Though Gall truly conceived the field and made it noticeable to both the public and to people of science, it was Spurzheim who popularized the theory to the masses of America and Europe, demonstrating how the field could be applied beyond scientific inquiry, such as the betterment of society and individuals via sexual partner selection and the determination of societal leaders [17,18]. Due to the works of the field’s later disciples, such as the brothers Orson Squire (1809-1887) and Lorenzo Fowler (1811-1896) in the United States and later England, the field was transformed from a system for understanding the complexities of human behavior and their supposed physical clues to a form of societal and individual self-improvement. Through the use of cranial casts, detailed charts, and published phrenological journals, organs of human faculties could be located, analyzed, and theoretically measured to create what proponents regarded as a more perfect, humane, or, in some cases, racially "pure" society or family.

One societal realm that often utilized phrenology was gender studies, which greatly benefited from the scientific community's largely recognized rejection of the field by the mid-19th century. Due to this rejection, much of the restrictive professionalism and gender codes that had plagued mainstream science for ages were absent in phrenology [16]. Though the field drew intrigue and enthusiasm from both men and women in 19th-century England and America, women in particular gravitated towards it because it encouraged a quest for self-knowledge and individual betterment, as well as a deeper analysis of the role of gender in a heavily patriarchal society. Differences in skull shape were used to reaffirm widely accepted gender norms in 19th-century America and Europe, often highlighting the perceived privileged and superior qualities of males and profiling female deficiencies in character [19]. Despite its reinforcement of this misogynistic ideology, the field also claimed to display some variability and similarities between the two sexes, thus blurring the definition between the behavior and capabilities of a typical male and female. For this reason, some women saw phrenology as a form of female empowerment, as they now had what they considered scientific evidence to support their feminine attributes while also safely claiming traits deemed masculine and thus considered favorable by 19th-century society, all without compromising their womanhood [19].

Aside from women, phrenology was also used for the advancement of racial groups, particularly Black people. Though phrenology was sometimes used to defend slavery, it was also used by some American abolitionists to expose the advantageous potential of the field for Black people as well as to highlight the sheer barbarism of slavery. After submitting himself to an examination by the Fowlers, the Black abolitionist William Cooper Nell (1816-1874) reported that the Fowlers found him to be "a man of integrity" who had "an excellent memory," was "high-minded, dignified," and aspired "to do something worthy of himself" [20]. Using findings like these, the abolitionist movement was able to use the field of phrenology to critique the perceived differences in intellect and personality between White and Black people, thus using supposed empirical evidence to dethrone the long-held racist beliefs concerning the two races. Not only that, abolitionists used the field of inquiry to highlight the hypocrisy of something as ancient and primitive as slavery in a society so morally and intellectually enlightened as what was perceived in 19th-century America and Europe. Even George Combe (1788-1858) of the Edinburgh Phrenological Society, a figure known for his numerous treatises proclaiming the significant biological differences between White and Black people, wrote to the American abolitionist Maria Weston Chapman (1806-1885) with the following question: "How is it possible for a people so moral, religious, enlightened, and free to defend and practice slavery?" [21]. Additionally, it can be said that phrenology provided Black people and abolitionists a supposed scientific basis to promote the moral necessity of ending slavery, as well as a way of detecting and improving the perceived mental deficiencies of racial minorities.

From its inception, the phrenological movement was met by staunch criticism. Oftentimes, such supposed evidence in support of the field's methodology was based on offensive social stereotypes or factitious reasoning, standards that were even unorthodox by early 19th-century scientific standards. As for its societal effect, though some did utilize the field as a medium for racial or female empowerment and increased self-awareness, as with other fields of science during that era, it was also used to endorse White supremacy and even enforce the necessity of imperialistic attitudes. By comparing cranial features of skulls from European colonies worldwide, phrenologists emphasized the proposed primitive nature of colonial subjects. They used their supposed scientific evidence to endorse the need for imperialistic policies across European nations [22]. In an 1863 text by John William Jackson of Edinburgh University, it was argued that Black people were an "utterly helpless child" compared to Caucasians, whom he considered to be "perfect man" [23]. As a result, those like Jackson preached the necessity of European support and supervision over their colonial subjects, indicating their perceived primitiveness and natural incapacity to care for themselves and thus echoing the cries of imperialism across continental Europe.

In the United States in particular, phrenology was used to propagate racist views of Black people and Native Americans to justify the start and continuation of numerous malicious social policies. Concerning slavery, American physician Charles Caldwell (1772-1853) famously analyzed the skulls of various ethnic groups in the 1830s. He noted that "by original organization and therefore radically and irredeemably…the African is an inferior race" compared to Caucasians and that their enlarged "animal organs" located at the back of their skulls rendered them unfit for freedom [21,24]. From this, Caldwell and others like him came to believe that phrenology provided empirical and undeniable evidence that Black people were in their rightful place as slaves and that Black people required a Caucasian supervisor to survive, going so far as to argue that "the Africans must have a master" [21]. As for Native Americans, Samuel George Morton's (1799-1851) Crania Americana and its detailed phrenological examinations and findings supposedly demonstrated that the "American Race" possessed a mind "averse to cultivation" and "slow in acquiring knowledge," presenting their mental capabilities as that of "continued childhood" and thus not conducive to the industrialized progress of early-19th-century America [25]. To Morton and others like him, these mental differences were seen as natural phenomena dictated by divine intervention and thus were incapable of being modified by one's own actions, contrary to the works of Spurzheim [26]. Thus, much like how phrenological works were used to justify the enslavement of Black people, they were also used to support Indigenous removal and resettlement policies in the United States during the 1830s and 40s, thus allowing for the westward migration of White settlers as well as their values and ideology.

Aside from enforcing scientific racism, the field of phrenology was also used as a weapon for propagating the superiority of certain social classes. Middle-class Caucasian women in particular used phrenological methodology to analyze their own values and personalities compared to other groups of people, most notably racial minorities, criminals, those of the lower classes, and those with physical disabilities [19]. With this revolutionary new form of quantitative, and thus objectively undeniable, evidence for evaluating behavior, men and women in one class could rightfully look down on others. Additionally, as the application of biology and medicine for the sake of societal good became more apparent in the 19th century, phrenological findings began to be used to promote women's health for the good of "the race," thus aligning with the fundamental ideals of the emerging eugenics movement [19]. Thus, while empowering some and allowing for the realization of societal equality for others, many during this time viewed the field as a form of scientific racism that had no place as a legitimate field of scientific inquiry. As a result, it is no surprise that, as early as 1815, phrenology was dismissed by one reviewer as "a piece of thorough quackery from beginning to end" [27]. Even the great American abolitionist Fredrick Douglass (1818-1895) commented on the racial aspect of phrenology, expressing that the famous phrenologist Morton depicted Europeans with "the highest ideas of beauty, dignity, and intellect" while also proclaiming "negro imbecility and degradation" [28].

Scientific discreditation of phrenology

Though chastised by the prominent and the lay alike for its malicious applications to social issues, no criticism was harsher than that from the scientific community regarding the field's legitimacy. From its inception, phrenology was under the close scrutiny of the broader scientific community and even the nobility of Europe. In France, Gall and Spurzheim's work was well received, though phrenology's status as a legitimate field of study and its followers' status as serious scientists were still lacking. To fix this, the pair submitted an account of their findings and the originality of their brain dissection methods to the National Institute of Arts and Sciences in 1808 [2]. Despite the field's growing popularity, the National Institute denied its legitimacy, suggesting that the great biologist Georges Cuvier (1769-1832, Figure 4A) had a role to play. While a commission headed by Cuvier did approve several neuroanatomical studies claimed by the trailblazer duo, such as the origins of the chief cranial nerves, the significance of the neural commissures, and the course of the pyramidal tract for motor function, the commission refused to endorse or even discuss certain findings and principles of Gall's organology doctrine [2]. When it came to the discussion of Gall's concept of cerebral localization and its relation to cranial prominences, the commission refused to even partake in such a discussion.

**Figure 4:**
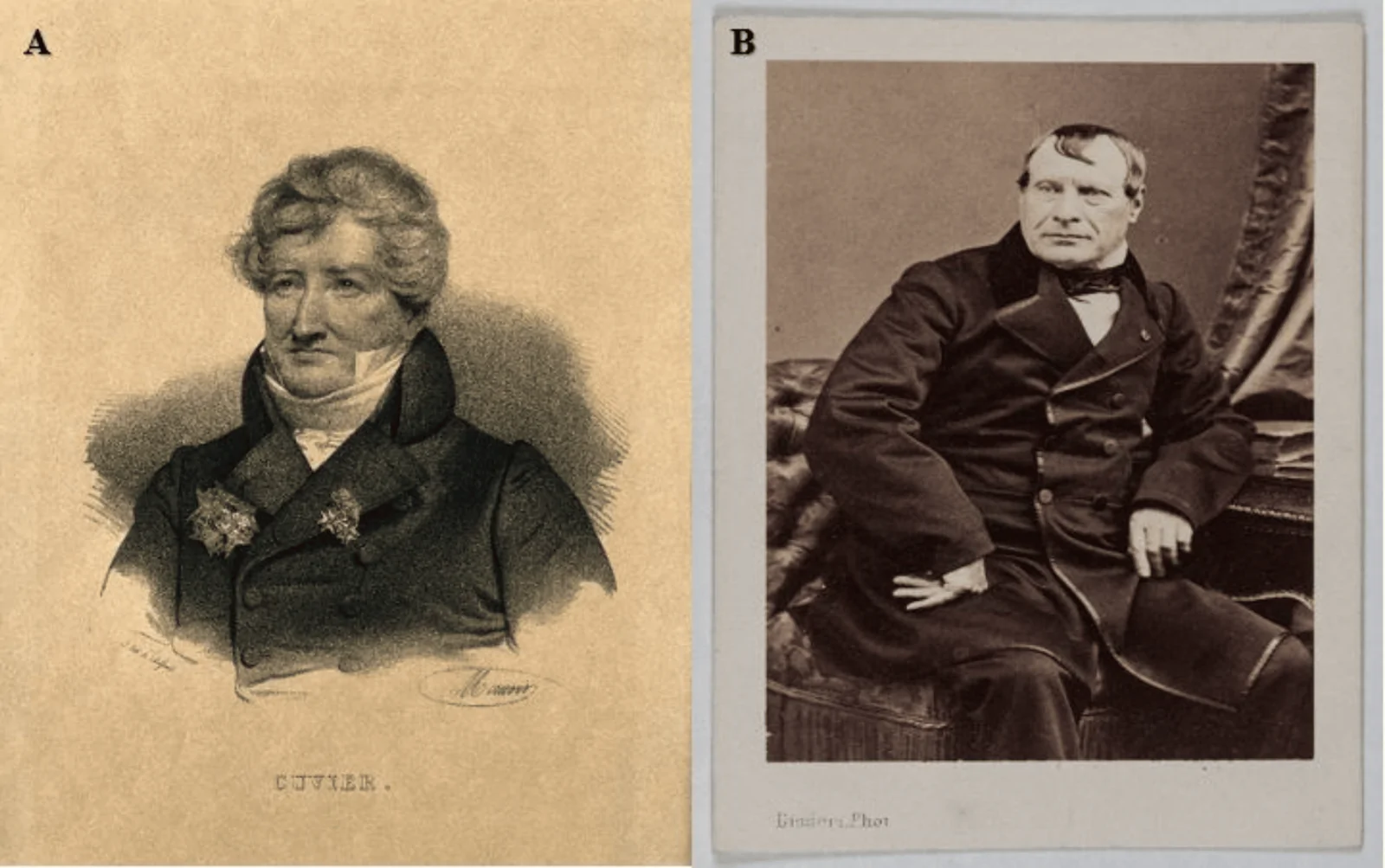
(A) Georges-Leopold-Chretien-Frederic-Dagobert, Baron Cuvier (lithograph by N. E. Maurin). (B) Marie Jean Pierre Flourens (photograph by A. Disderi, 1867) Image Credit: (A) Wellcome Collection. Public Domain Mark. Licensed under Creative Commons Attribution 4.0 International (CC BY 4.0): https://wellcomecollection.org/search/images?query=trt8krkm (B) Paris Musées/Musée Carnavalet (CC0 1.0 Public Domain Dedication), accessed via Wikimedia Commons: https://commons.wikimedia.org/wiki/File:Portrait_de_Pierre,_Jean,_Marie_Flourens_(1794-1867),_(physiologiste),_PH50650_(1_of_2).jpg

Despite the duo's eventual success of their five-volume work, the scientific community of France and even England continually refused to accept the field, resulting in attacks from numerous experimental physiologists who sought to disprove the findings of Gall and Spurzheim. Eventually, even the National Institute commissioned its own investigators to assess the validity of Gall's theory, among them the famous French physiologist and Cuvier protégé, Marie-Jean-Pierre Flourens (1794-1867, Figure 4B). Working in conjunction with and supported by the Cuvier commission, Flourens became one of the loudest critics of Gall's cerebral localization doctrine. They strongly criticized phrenology and its supporters in numerous publications [29-31]. Though Flourens demonstrated in 1822 that the cerebellum is associated with motor coordination and that the cerebral cortex is associated with intelligence, sensation, and perception, he found nothing to suggest that specific regions of the brain, or the cerebral cortex in particular, were responsible for specific mental characteristics [32]. Despite protests from Gall, Flourens's work had the support of the National Institute and, more specifically, Cuvier, thus almost entirely silencing the concept of cerebral localization for nearly the next 40 years [2].

Despite rebuttals from Gall and Spurzheim, the publicity surrounding Flourens's findings caused momentous damage to the phrenology movement, marking the beginning of its inevitable end. Following Flourens's work, other clinical observers devised their own experiments in hopes of replicating phrenological findings, with many failing to do so and publishing their results afterward [33]. Following this, phrenology's followers began to distance themselves from the movement, with some even suggesting they had never truly embraced the field's principles in the first place [34]. By the 1840s, aside from the general public, phrenology as a field of legitimate scientific inquiry was nearly completely forgotten. So much so that the famous French physiologist Francois Magendie (1783-1855) commented in an 1844 treatise that phrenology was "a pseudo-science of the present day," likening it to "astrology, necromancy, and alchemy of former times" [35].

Phrenology and its lasting impact

By the 1840s, the field of phrenology had mostly been discredited in the lecture halls and laboratories of academia. The idea that an individual could determine the personality, memories, and emotions of another simply by the features of their cranium had now been thought to be unreliable, not factual, and, to put it mildly, nonsensical. Despite this, the question that led to the creation of the pseudoscientific field in the first place remained: are aspects of the human psyche truly specific to distinct anatomical regions of the brain? Or is all human thought, intellect, and emotion simply the result of widespread neural activity and processing involving multiple, distant regions of the brain simultaneously? This question of cerebral localization or widespread cortical involvement remains as much an area of significant debate in the field of neuroscience today as it was for 19th-century neuroscientists. Though applied incorrectly in phrenology, it was this very question that prompted investigations by prominent researchers and later titans within the field of brain science, such as Paul Broca (1824-1880), David Ferrier (1843-1928), and Carl Wernicke (1848-1905), and that would inspire future generations of brain researchers [36,37].

Despite these later unprecedented studies and their findings, this concept was perhaps most famously illustrated in the celebrated case of traumatic brain injury, which came to be known as the "American Crowbar Case" [38,39]. Following a head injury sustained at a worksite in Cavendish, Vermont, in 1848, 25-year-old Phineas Gage (1823-1860) began displaying abrupt changes in personality and behaviors that seemed to bewilder people familiar with the man [40,41]. Friends, family, and co-workers noted that the once-intelligent, motivated, and friendly Gage had become an obscene, irresponsible, and socially uninhibited stranger following the accident [40]. Following this accident, Gage's "mind was radically changed, so decidedly that his friends and acquaintances said he was 'no longer Gage'" [42].

Aside from renewing the scientific community's interest in the concept of cerebral localization and later propelling the theory's future researchers into the annals of neuroscience, this case also illustrates a unique and feasible core principle in the thoroughly debunked and widely rejected theory of phrenology. From this case, the idea that specific regions of the cerebrum could be responsible for facilitating specific aspects of human behavior became a feasible hypothesis, as Franz Gall had proposed decades before the influential works of Flourens, Broca, and Wernicke. In the end, like many theorists and thinkers before and after them, the fundamental concepts of Gall's organology and Spurzheim's phrenology contained a limited element of scientific validity, which was later supported and widely accepted by prominent members of the scientific community. Through later investigations by Broca, Wernicke, and Ferrier, areas of the brain responsible for language, motor function, sensation, and visual processing were eventually identified and extensively studied, thus confirming a variant of Gall and Spurzheim’s concept of the compartmentalization of human behavior. Though initially flawed and based on flimsy evidence even by 19th-century scientific standards, clinical studies would later support Gall's underlying doctrine of cerebral localization, with some recognizing this as his chief contribution to the field of neuroscience and humanity's centuries-long quest to understand the ever-perplexing nature of the human brain [2].

## Conclusions

The case of Gall and Spurzheim's phrenology illustrates the complexities of science and the uncertainty of proposed scientific theories, whether they are initially widely popular or universally disapproved. The study of phrenology serves as an archetype of the infinitely uncertain nature of scientific thought and theory, as well as how newly presented evidence may upend centuries-held views of the natural world or resurrect theories or concepts that were once regarded as nonsensical or inconceivable. However, like countless other fields of inquiry before and after Gall and Spurzheim, the anecdotal evidence supporting their fundamental views, the malicious application of their field to societal roles and classes, and the rejection and empirically based disapproval of the field eventually led to its slow death. This ultimately cast the maverick field of phrenology into the annals of history as perhaps the embodiment of the term 'pseudoscience'."
